# A systematic exploration of differences in contextual factors related to implementing the MOVE! weight management program in VA: A mixed methods study

**DOI:** 10.1186/1472-6963-11-248

**Published:** 2011-09-30

**Authors:** Laura J Damschoder, David E Goodrich, Claire H Robinson, Carol E Fletcher, Julie C Lowery

**Affiliations:** 1 Ann Arbor VA Center for Clinical Management Research, 2215 Fuller Road, Ann Arbor, MI, USA

## Abstract

**Background:**

In January 2006, Veterans Affairs (VA) disseminated the MOVE!^® ^Weight Management Program to VA medical centers to address the high prevalence of overweight/obesity. In its second year, MOVE! implementation varied widely across facilities. The objective of this study was to understand contextual factors that facilitated or impeded implementation of MOVE! in VA medical centers in the second year after its dissemination.

**Methods:**

We used an embedded mixed methods cross-sectional study design. Qualitative and quantitative data were collected simultaneously with the primary purpose to explore contextual factors most likely to influence MOVE! implementation effectiveness at five purposively selected facilities. Facilities were selected to maximize variation with respect to participation in MOVE! by candidate Veterans. Semi-structured phone interviews were conducted with 24 staff across the five facilities. Quantitative responses were elicited followed by open-ended questions. The quantitative measures were adapted from a published implementation model. Qualitative analysis was conducted using rigorous content analysis methods.

**Results:**

Qualitative and quantitative data converged to strengthen findings that point to several recommendations. Management support can help increase visibility of the program, commit needed resources, and communicate the importance of implementation efforts. Establishing a receptive implementation climate can be accomplished by emphasizing the important role that weight management may have in reducing incidence and severity of obesity-related chronic conditions. Coalescing highly functioning multi-disciplinary teams was an essential step for more effective implementation of MOVE!. In some situations, local champions can overcome challenging barriers in facilities that lack sufficient management support.

**Conclusions:**

Key organizational factors at local VA medical centers were strongly associated with MOVE! implementation. Results pointed to recommendations that can help accelerate large-scale dissemination of complex weight management programs.

## Background

The prevalence of overweight/obesity is at epidemic proportions in the adult population [1] and even higher among Veterans in the U.S. [2]. Nearly three-fourths of the 5.7 million Veterans [3] who receive their medical care from the Veterans Health Administration (VHA) are overweight or obese [2]. Overweight and obesity are associated with substantial morbidity and mortality [4-7] and increased healthcare costs for patients, healthcare systems and payers [6,8,9]. In 2001, VHA primary care providers cited effective weight management programs as the most pressing need in preventive services for Veterans [10].

To address this need, the VA National Center for Health Promotion and Disease Prevention (NCP) developed, pilot tested and launched the MOVE!^® ^weight-management program in January 2006 under VHA policy that all facilities implement MOVE! or another comparable weight management program [10]. NCP designed MOVE! as a patient-centered, multi-tiered set of tools and treatment options based on evidence-based guidelines and tailored to individual needs [10-13]. MOVE! comprises five increasingly intensive treatments for weight management as shown in Table 1[14]. The types and amount of treatment provided at local facilities depend on the degree to which those facilities implement program components and on patient needs and preferences. All VA medical centers were asked to provide points of contact for a regional coordinator, local facility coordinator, and physician champion for MOVE!. NCP developed a comprehensive set of implementation guides for local facilities (many of which can be viewed publicly online: http://www.move.va.gov) and conducted monthly support conference calls with regional program coordinators who in turn, conducted calls with local facility coordinators. No funding was given to local facilities by NCP to implement this program.

**Table 1:** Description of VA MOVE! Stepped Care for Weight Managementa

Treatment Component	Description
Assessment	Multi-factorial assessment of food and beverage intake, physical activity habits, as well as personal and family history, self-efficacy, self perceptions, and readiness to change with regard to weight management (e.g., using the 23-item MOVE!23 questionnaire)

Individual or group self-management support	On-going multi-disciplinary group meetings with individual consultation as indicated

Pharmacologic Agents	Addition of pharmacologic agents

MOVE! Intensive	Medically-intensive weight management interventions

Bariatric Surgery	Bariatric surgery; follow-up care

a http://www.move.va.gov

In 2006, VHA had a network of 155 medical centers and 872 community-based outpatient clinics [10]. Currently, dissemination of the MOVE! weight management program in VHA is the largest and most comprehensive dissemination of a weight management program in the U.S. Nearly 300, 000 Veterans have participated in MOVE! from its roll-out in January 2006 through January 2010, generating 1.7 million visits. Veterans are candidates for MOVE! if their body mass index (BMI) is more than 30 kg/m^2 ^or between 25-30 kg/m^2 ^with one or more obesity-related chronic health conditions e.g. hyperlipidemia [10].Despite the volume of participants and services provided, the number of MOVE! Veterans who would most benefit from this program is so large that of 1, 000 Veterans who were candidates for MOVE!, about 29 received care through MOVE! in Fiscal Year (FY) 2010.^i ^Though reach of this program is relatively low in a given year, this rate represents a 3.3-fold increase in the number of Veterans who participated in MOVE! annually compared to the first year of the program when only about 8 per 1000 candidate Veterans participated. In the second year of the program, local facilities varied widely in the number of candidate Veterans who participated in MOVE!; from no participants at many facilities to a high of 37 participants per 1000 MOVE! candidates.

The aim of this study was to identify differences in organizational factors between facilities with effectively implemented and relatively robust MOVE! programs and those with poorly implemented programs. We hypothesized that facilities with effectively implemented MOVE! programs would show differences in quantitative and qualitative measures of key organization constructs compared to facilities with poorly implemented programs. A secondary aim was to explore the validity of our quantitative measures of organizational factors. Findings from this study can be used to help develop recommendations to guide future large-scale disseminations of multi-component weight management programs like MOVE!.

## Methods

We used an embedded mixed methods cross-sectional study design [15]. Qualitative and quantitative data were collected simultaneously during participant interviews, with the primary purpose to explore contextual factors most likely to influence MOVE! implementation effectiveness at the study facilities.

### Implementation Framework

We adapted a model developed by Klein and colleagues that specifies hypothesized antecedents to complex implementations [16]. This model has been tested empirically in a large sample of manufacturing facilities that had all purchased and implemented the same software technology [17]. Helfrich and colleagues applied and expanded this model in a qualitative study of four cancer clinical research networks and found that the model helped explain differences in implementing cancer prevention and control research across multiple sites. This model is unique in its applicability to *complex *implementation efforts, like MOVE!, which require intentional, coordinated use by multiple organizational members across service lines to implement and achieve expected benefits [17,18].

**Figure 1:**
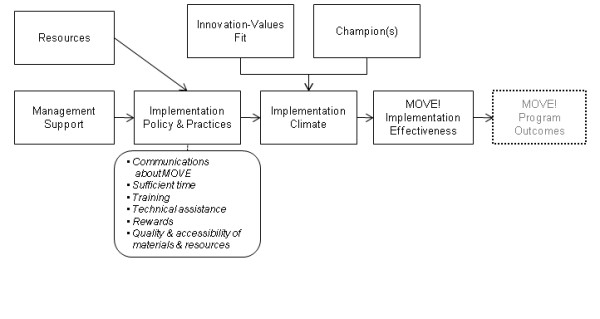
**Theoretical Model for Complex Implementation**.

The implementation model, as adapted for our study, is shown in Figure 1. Constructs included in the model are based on Helfrich and colleagues' expanded version of Klein and colleagues' model [18]. In this model, the unit of analysis is the medical center. Thus, model constructs are evaluated in terms of the collective, summary perception by targeted staff at each facility. The following is a summary of definitions for each construct [16-18]:

#### Management Support

The extent to which managers are committed and willing to invest in and to monitor the quality of implementation policies and practices.

#### Resources

Availability of financial resources to fund staff, computers, etc., including facility space.

#### Implementation Climate

Employees' shared perceptions of the importance of program implementation within the organization.

#### Implementation Policy and Practices

A broad construct related to organizational policy and practices that may influence implementation, including: 1) quality and quantity of training; 2) technical assistance on an as-needed basis; 3) rewards, including promotions, praise, or improved working conditions; 4) effective communication about the goals of the implementation; 5) sufficient time for users to experiment or learn new skills related to the innovation; 6) quality, accessibility, and user-friendliness of the innovation itself.

#### Innovation-Values Fit

The extent to which targeted users perceive that use of the innovation will foster (or, conversely, inhibit) the fulfillment of their values.

#### Champions

Champions promote the innovation with targeted organizational members and management.

### Outcome Measure

MOVE! implementation effectiveness is our study outcome, operationalized as the relative proportion of candidate Veterans who participated in MOVE! and whether the treatment components for individual and/or group self-management support existed (see Table 1). It was outside the scope of this study to evaluate MOVE! program outcomes (e.g., weight loss among Veterans).

### Facility Selection

Five VHA medical centers were selected using a purposive selection process congruent with our study aims. NCP provided the study team with a list of 18 facilities that reported extremely high or low numbers of MOVE! visits and that were not already participating in a MOVE!-related research study. The study team ranked this list of facilities based on a projected number of patients (because facilities were selected in the middle of FY2007) who would receive at least one MOVE! visit in FY2007 as a proportion of the estimated number of MOVE! candidate Veterans. This ratio provided a preliminary indication of the relative implementation effectiveness of MOVE! across facilities. The study team chose 3 facilities with the highest rates of Veteran participation and 2 facilities with the lowest rates of participation. These facilities were later confirmed to be in the highest and lowest quartile of facilities in VHA for FY 2007 based on actual data for the FY. Facilities were purposively selected within the highest and lowest quartiles to maximize diversity [15] with respect to geographic location and types of MOVE! visits that were reported (some facilities reported only individual visits, some only group visits, and some facilities reported both types of visits). See Table 2 for study facility characteristics.

**Table 2:** Characteristics of VA Study Facilities

Implementation Effectiveness & Rationale	Number of MOVE! Participants/1000 Candidate Veterans FY2007	Number and Type of MOVE! Visits FY2007	Active MOVE! Treatment Components
**Low Implementation** Despite top-quartile reported level of participation, Veterans receive only an initial assessment, then are referred to a community-based program with no follow-up. Rate of participation dropped significantly to 12.3 in FY2008.	26.7 (Top Quartile)	Individual: 259 Phone: 0 Group: 3	• Initial assessment; no self-management support

**Low Implementation** Initial assessment and group visits are present but participation continues to be among the lowest because of challenging organizational constraints. Participation remained low; 3.4 in FY2008.	3.8 (Bottom Quartile)	Individual: 0 Phone: 73 Group: 372	• Initial assessment; no individual phone-based self-management support • 10-week series of weekly group classes • "Reunion" group at end of each series

**Transition** After a first failed attempt, efforts were well underway by the time of our interviews. Participation rates increased to 7.1/1000 candidate Veterans in FY 2008.	0.4 (Bottom Quartile)	Individual: 0 Phone: 0 Group: 1	• Initial assessment; no self-management support • 6-week series of weekly group classes • Ad hoc post-completion support

**High Implementation** Top quartile in participation FY 2007. High use of initial assessments and group visits with ad hoc phone support. Participation remained high; 19.4 in FY2008.	19.2 (Top Quartile)	Individual: 984 Phone: 574 Group: 1773	• Initial assessment; limited self-management support • 10-week series of weekly group classes • Ad hoc post-completion support

**High Implementation** Top quartile in participation in FY 2007. The most comprehensive implementation of MOVE! among the study facilities. Program participation remained high; 37.7 in FY2008.	27.6 (Top Quartile)	Individual: 212 Phone: 349 Group: 2311	• Initial assessment; limited self-management support • 6-week series of weekly group classes • Therapy using pharmacological agents • Intensive outpatient lifestyle counseling program • Bariatric surgery

### Study Participants

Interviewees from facilities were recruited between July and October 2007. We first identified the regional and local facility MOVE! coordinators and interviewed them. Regional MOVE! coordinators provided support for the local MOVE! coordinators at the facilities in their region and were liaisons with NCP, the national VA office that disseminated MOVE!. We used a snowball sampling technique by asking the local facility coordinators to identify all staff who were involved with delivering or implementing MOVE! at their facility [19]. We invited all named staff members to participate in the study, continuing to ask for additional names to ensure we invited everyone involved. Individuals were invited by the study team via email which included a brief description of the study and the interview process. A waiver of signed informed consent was granted by the VA Ann Arbor Healthcare System Institutional Review Board. Participants were verbally consented at the start of the telephone interview; permission to record the interview was also obtained. Participants were told they could have the recorder turned off at any point in the interview and were offered a $10 gift card as a token of appreciation for their time. All interviews were digitally recorded and transcribed verbatim.

### Measures and Data Collection

Our objectives were to both identify differences in organizational factors between sites with high implementation effectiveness versus sites with low implementation effectiveness (the primary outcome depicted in Figure 1) and to explore the validity of our quantitative measures of those organizational factors. As described earlier, implementation effectiveness was deteremined based on the rate of Veteran participation in MOVE! and the existence of key treatment components. Veteran participation was calculated using data from system-wide decision support system databases. Active treatment components were identified for each facility from interview data.

The organizational factors affecting implementation effectiveness were assessed both quantitatively and qualitatively. Quantitative data were used as an efficient data collection mechanism; qualitative data were used to validate the quantitative data and to provide greater insight into local implementation processes. The quantitative measures were obtained from a questionnaire developed by Klein and colleagues [17]. We chose a subset of items that: 1) were most salient for this study; 2) required minimal rewording to apply to our context; and 3) were most dissimilar in content (i.e., we excluded items that appeared to be duplicative). We also collected qualitative data to further characterize each of the organizational factors of interest. The interview guide, including quantitative items is included in Additional File 1: Interview Guide.

The interviews opened with the 18 quantitative items with Likert-scale responses, which addressed the following constructs: communications (5 items; part of Implementation Policy and Practices), climate (7 items), management support (6 items), time availability (3 items; also part of Implementation Policy and Practices), and resources (7 items). Each item was presented as a statement (e.g., "I have access to a lot of information about MOVE!"), with 6 possible responses (*strongly disagree, disagree, neutral, agree, strongly agree*, or *unknown/not applicable*).

The quantitative items were followed by a series of open-ended questions. It was important to encourage open narration to elicit information the interviewee deemed important [20], rather than bias their responses by asking a series of questions that strictly followed the Klein et al. constructs. Thus, the interviews included questions about each respondent's role in the MOVE! program, their relationships with other professionals within and outside their facility, how they implemented MOVE!, etc. When we needed more information regarding, for example, a potential barrier, we asked follow-up questions to elicit more details.. Qualitative questions were adapted based on who was being interviewed, the time available for the interview, and previous information shared by other interviewees at the same facility. The principal investigator (LJD) led all the interviews. At least one other team member also participated to help ensure all topics were covered and to allow for more accurate coding of responses by a second person who heard the responses first hand.

### Qualitative Data Coding, Analysis, and Interpretation

We conducted content analysis [21] of our interview data, guided by a consensual qualitative research approach [22,23] and supported by NVivo8 qualitative analysis software. The consensual research approach has the following features: 1) data are collected through open-ended questions in semi-structured interviews as described above; 2) multiple judges are used throughout data analysis to foster multiple perspectives; 3) consensual validation is achieved through a process of deliberation and consensus [24]; 4) an outside auditor (an expert not integrally involved in the study) reviews the process to help maximize validity of findings; and 5) constructs are identified and applied to cases and cross-analyses are performed (comparing and contrasting cases and examining the data for patterns). The following paragraphs provide more detail.

Interview data were coded deductively according to each of the constructs depicted in Figure 1, as well as coded inductively according to additional themes identified from the data. Two teams of analysts (a health psychologist, a PhD nurse-researcher, a health education/health behavioralist, and a research assistant) worked in pairs. All four analysts coded the first few interviews, and then met to refine construct definitions and ensure a common understanding of constructs across analysts. Each team was assigned an interview and each person in the team reviewed and coded each transcript independently. Each pair of analysts met and used a consensus process to write and build on summary statements along with supporting quotes for each facility. The analysts were rotated week by week so that each had a different partner to help strengthen validity of findings by encouraging analysts to face conflicting views with different partners each week, critically test, analyze, and evaluate coding and summaries (to avoid "group-think"); and to encourage balance in terms of power and participation in discussions [25].

Each pair of analysts presented its summaries to a larger team consisting of the two pairs of analysts, the study PI (LJD) who had implementation and qualitative research expertise, another researcher with implementation research expertise (JCL), and a qualitative expert. The qualitative expert acted as an auditor by providing a neutral check to help ensure consistent application of our approach. The implementation researchers provided two additional perspectives with one being intimately familiar with the data, having conducted the interviews and reviewed and analyzed the transcripts (LJD) and the other regularly questioning assumptions and conclusions to help ensure strong rationale for coding and summary statements (JCL). "Analyst triangulation" (where two or more people independently analyze the same data and compare findings) [26] occurred at two levels: within the pairs of analysts and within the larger team to help reduce the possibility of bias. The large team of seven analysts and researchers convened weekly until all the interview transcripts were coded and analyzed.

This process resulted in five documents, one for each facility, with summaries and quotes for each construct within each facility along with an overall summary statement. The summaries included a description of MOVE! treatment components for assessing implementation effectiveness.

### Quantitative Data Analysis

The unit of analysis for the five quantitatively measured constructs was the facility. Thus, scores were averaged across individuals within each facility. Cronbach's alpha, which assesses internal scale consistency reliability, ranged from 0.73 to 0.85 across the five constructs at the individual level. Sample size was not sufficient to compute alpha at the facility level. Average scores for each construct are presented by facility. Quantitative data were analyzed using Stata V11 [27].

## Results

### Participants

Of the individuals who were identified as having a role in MOVE! implementation, 75% (n = 24) agreed to participate. Table 3 lists participant characteristics. Of note, only 40% of physician champions agreed to be interviewed; insufficient time was their chief reason for declining.

**Table 3:** Participant Characteristics

Role of Participant	Low Implementation	Transition	High Implementation
Regional Coordinator^a^	2	1	2

Facility Coordinator	2	1	2

Physician Champion	1		1

Physician		1	

Nursing			2

Food & Nutrition	1	1	2

Physical Therapy			1

Mental./Behavioral Health	1	1	1

Other	2		

**TOTAL**	**9**	**5**	**11**

**Participation Rate (%)**	70	63	84.5

a. One regional coordinator is counted twice because 2 facilities are in the same region.

### Implementation Effectiveness

Table 2 shows the rated effectiveness of implementation for each of the five facilities along with the rationale for each of the ratings, participation rates, number and types of MOVE! visits reported, and MOVE! treatment components that were implemented and actively running at the time of the interviews. Of the three facilities with MOVE! participation rates in the top quartile, two also had several treatment components of the MOVE! program, including initial assessments, self-management support, group classes, and some degree of follow-up support, thus earning them the designation of "high implementation effectiveness" sites. However, one of the sites with participation rates in the top quartile had implemented only one treatment component of MOVE!--the initial self-assessment. Confirmation of the tenuousness of this program was reflected in significantly lower participation rates in the following year (FY '08). Therefore, this site was placed in the "low implementation effectiveness" category. Of the two facilities initially selected because of low MOVE! participation rates, one remained a "low implementation effectiveness" site because there was no improvement in their rates of participation and because they had not implemented one of the treatment components--phone-based self-management support. In contrast, the other facility with a low initial participation rate significantly increased participation between FY '07 and FY '08 and, therefore, was labeled as a "transition" site.

### Organizational Factors

**Figure 2:**
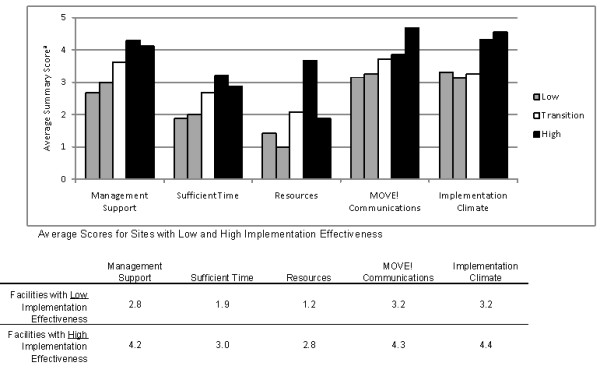
**Quantitative Measures of Model Constructs (n = 5)**. a. 1-5 scale; a. 1 = Strongly Disagree to 5 = Strongly Agree; some items were reverse-coded so that lower values indicate poorer ratings (see Additional File 1).

Qualitative and quantitative data both showed clear differences between facilities with high versus low implementation effectiveness. Quantitative measures from the transition facility generally had values that fell between the facilities with high and low implementation effectiveness. The following sections present results, organized according to our guiding theoretical model with qualitative data presented first followed by quantitative results when available. Figure 2 compares quantitative scores for each construct across the five facilities.

#### Resources

Constrained monetary funding was only mentioned by interviewees at the two facilities with low implementation effectiveness. As one interviewee said,

"Well there's nothing like an unfunded mandate...to get...their blood boiling around here where workloads are so high everywhere else." [MOVE! Team Member]

At the other low implementation facility, the MOVE! coordinator struggled with many constrained resources including lack of money to support the program.

Our coordinator's extremely distressed over facility issues and begging...management has not put money forth for things. [MOVE! Team Member]

Until the facility director finally approved funds, the MOVE! coordinator purchased supplies out of her own pocket as incentives for Veterans who completed the MOVE! classes.

Dedicated staff time was a frequently mentioned resource constraint along with lack of space. Space constraints presented a challenge, especially at one facility with low implementation effectiveness. Here, launch of MOVE! was delayed by several months because they could not find space in which to conduct group visits:

We had wanted to start earlier in the spring [sic]. There was a lot of conflict with scheduling. The room was only available certain times of the day and it conflicted with other group classes in the room...we basically moved into a room that was full of storage and we offered to go in there and try to make it conducive to a classroom and once we showed that there was going to be some attendance and it was going to be an ongoing and successful project we were able to get a more permanent location. [MOVE! Coordinator]

Quantitative measures of resource availability reflected that though none of the facilities received extra funding to implement MOVE!, constraints were more strongly felt (a difference of 1.6 on a 5-point agreement scale) in the facilities with low implementation effectiveness versus those with high implementation effectiveness; the transition facility had a score in between the low and high implementation facilities, reflecting the mixed experiences as they transitioned into new leadership for MOVE!.

#### Management Support

Regional and local management support at both of the facilities with high implementation effectiveness was strong as evidenced by their active efforts on behalf of MOVE!. For example, regional coordinators lobbied for dedicated staff and training at local facilities and helped promote MOVE! visibility by getting it on key meeting agendas:

[Regional leaders] continue to look [out] for us. If you say 'Can you bring it up at this meeting'... they certainly will, " [Nurse Supervisor].

MOVE! implementation required a multi-disciplinary team. Management support across multiple units was needed because team members reported to different supervisors who, at the high-implementation facilities, were generally receptive because top leaders made it clear that they wanted the program implemented "by a specific time."

Both facilities with low implementation effectiveness suffered from an absence of management support. One low-implementation facility implemented MOVE! through largely grass-roots efforts without management support or commitment to MOVE! as evidenced by the absence of fundamental resources needed to implement MOVE!. One supervisor did not even know of her subordinate's involvement with MOVE!.

The story of implementation at the transition facility highlights the significant influence that managers can have on implementation. The facility director appointed a physician champion and a MOVE! coordinator but that seemed to be the extent of attention from leadership in the first year. Program implementation was regarded as a failure during MOVE!'s inaugural year at this facility. We interviewed staff shortly after a major change in MOVE! leadership, which was a significant turning point in program implementation. A service line chief leveraged her temporary appointment as acting facility director:

...I said, 'you're either going to do it or you're not and you're going to have to pay a dietitian to come in...' [and] the Veteran Service Organizations, it was in the spotlight for them too....the timing couldn't have been more perfect... [Service Chief]

This leader was able to gain commitment from facility leaders for dedicated staff including hiring a dedicated MOVE! coordinator who launched a large pilot program with support from this manager.

The substantial difference observed in management support across facilities with high and low implementation effectiveness was reflected in the quantitative measure for this construct. Figure 2 shows that facilities with low implementation effectiveness had the lowest scores for management support. The average score for the two facilities with high implementation was 1.4 points higher (on a 5-point scale). Not surprisingly, the transition facility had a score in between the low and high implementation facilities.

#### Implementation Policy & Practices (IP&P)

We found no differences in facilities' experience with training (no one talked about receiving formal training), technical assistance (most of the facilities relied on resources available online and ad hoc communications with their regional coordinator), quality and accessibility of materials and resources (most of the facilities relied on these materials to develop their programs and found them very useful), or rewards (no rewards or incentives were in place for implementing MOVE!). However, time constraints and communications about MOVE! differed between facilities with high versus low implementation effectiveness.

##### Sufficient Time

Staff time was highly constrained at all of the facilities. Phone-based self-management support was not offered or was very limited in all of the facilities because staff: 1) lacked the time to follow up with patients using recommended protocols; or 2) lacked confidence in their ability to provide effective support over the phone.

Limited staff time constrained their ability to meet burgeoning patient demand even at the high implementation facilities. These facilities had enrolled a very small proportion of candidate Veterans and yet already had long waiting lists:

It's obvious that all my sites have somewhat maxed out their capacity so they really can't run the programs any higher...we're not going to be able to grow the program and it's not where it should be yet. [Regional Coordinator]

One high implementation facility coordinator reluctantly had to ask a patient to stop recruiting fellow patients into the program:

...one of our patients...goes and talks to other patients in the waiting rooms saying what a great program it is...[but] we actually can't really accommodate now because of the time constraints... [MOVE! Coordinator]

Quantitative measures of sufficient time reflect differences (the measure averaged 1.6 points lower for facilities with low implementation effectiveness versus high implementation). This score indicates that time constraints are felt more acutely at facilities with low implementation effectiveness.

##### Communications

The visibility of MOVE! at the high implementation facilities, at least in part enabled through effective communications about the program, fostered a robust rate of referrals from primary care providers at the high implementation facilities. In contrast, communications about MOVE! were poorer at the low implementation facilities. We heard stories of patients at both of the low implementation facilities approaching program staff with confusion, thinking they were going to see a movie, be in a dance class, or that they were being referred for bariatric surgery.

One outcome of highly effective communications was the coalescing of the MOVE! teams at the high implementation facilities. The MOVE! teams included the facility coordinator, a behavioral psychologist, a dietician, and sometimes a physical therapist. Teams at the two high implementation facilities met weekly or bi-weekly to problem-solve, review cases, and coordinate and refine presentations for classes, building a shared sense of purpose and mutual respect:

... there isn't a week that doesn't go by that...we're not communicating with each other and...we're having a good time too with the group sessions [MOVE! Team Member]

Staff at the low implementation facilities did not coalesce as a team. Program meetings and communications were irregular and the team, "just [met] through email." Staff from other departments led their assigned sessions but that was the extent of their participation in the program due to competing clinical duties in their own service.

Another aspect of communications that arose inductively from the data and was not captured by our operationalized definition of communications, was the influence of the general quality of communication across functional units. For example, the MOVE! coordinator at one high implementation facility worked and communicated closely with primary care providers which enabled medications to be adjusted for patients who lost weight and whose blood sugar levels improved, while delaying the start of new medications when appropriate.

The quantitative measure of MOVE! communications shows a trend similar to the other measures. Facilities with high implementation effectiveness had an average score 1.1 points above the two low implementation facilities. The score for the transition facility fell in between.

#### Innovation-Values Fit

Both of the facilities with high implementation effectiveness had components of weight management services in place prior to MOVE! dissemination making MOVE! highly compatible with existing programs. In one high implementation facility, MOVE! was viewed as a means by which to expand their program and make it even more visible among providers and patients. MOVE! aligned with clinical values in that providers at both high implementation facilities embraced lifestyle change as an important approach for weight management to reduce risk factors like high blood pressure:

I would say 99.99%...of the providers recognizes that [obesity] is in some way hindering their success in managing diabetes or managing blood pressures or managing hyperlipidemia... So everyone is very receptive...to refer the patients to MOVE! [MOVE! Coordinator]

Providers at these two facilities were clearly: 1) aware of the association between overweight/obesity and chronic conditions (e.g., diabetes); 2) cognizant of high overweight/obesity prevalence among Veterans; and 3) perceived MOVE! as a key strategy for addressing these concerns. The MOVE! program aligned with their desire to provide weight management treatment to Veterans and this helped provide impetus to approve additional dedicated personnel for MOVE!, though they were not yet in place at the time of our interviews:

...[what sold them was] the huge volume of patients that are eligible for treatment and care and at present...we're still not even reaching 1% of those patients...When you're trying to do this in your spare time, and there's no staff, you're in trouble. [Regional Coordinator]

In contrast, at the two low implementation facilities, MOVE! did not align with stated values. At one low implementation facility, one reason they referred patients to the community-based program was their perception that MOVE! was a weight loss program for obese Veterans, depicted by "not very positive pictures [of] depressed looking, heavy sailors in stretched out white tee-shirts, " in contrast to the community program which focused on wellness. At the other low implementation facility, many providers did not seem to believe that MOVE! would be successful in reducing patients' chronic disease risk factors.

#### Champions

Every facility was asked to identify a physician champion. Most of the facilities in our study had them appointed by the facility director. Physician champions at three of the study facilities (one high implementation facility, the transition facility, and one low implementation facility) did not have an active role in MOVE!. Two physician champions, one located at a high implementation facility and one at a low implementation facility, did actively help overcome barriers and provided moral support for their facility's MOVE! coordinator. The physician champion at one low implementation facility was especially active in working with the MOVE! coordinator; together they managed to get the program up and running in a very challenging environment.

#### Implementation Climate

Implementation climate at one high implementation facility was strengthened because the MOVE! coordinator was able to link implementation of MOVE! to their high-priority bariatric surgery program:

"...we were approved to start a bariatric surgery program bam, right away...all resources and interests [were] funneled into bariatric surgery...we did everything backwards...In hindsight, it probably was a good way to do that because our criteria for eligibility for people to have bariatric surgery is that they must be enrolled in MOVE! for one year." [MOVE! Coordinator]

The implementation climate for MOVE! was clearly lower at the two low implementation facilities. At one facility, MOVE! had lower importance than other pressing issues at that time because "staff work[ed] weekends and lunches to get through the backlog of patients in primary care." Furthermore, at this facility, the pressure to meet goals related to national performance measures worked at cross-purposes with implementing MOVE!:

...generally in this [region], everything takes a backseat to performance measures. If something isn't a performance measure or figures into the performance appraisal for the [regional] director and that facility director, then it isn't given the same weight...I think it's the number one barrier [for MOVE! implementation]...[Regional Coordinator]

The quantitative measure of implementation climate was markedly higher at the facilities with high implementation effectiveness compared to the low and transition facilities.

## Discussion

Consistent with our theoretical model, results support the role of management support, resources, implementation policy and practices, the degree to which MOVE! fits with values and existing programs, and the implementation climate as antecedents to MOVE! implementation. The importance of the role of champions was mixed.

Administrative and clinical management at the regional and local levels actively supported efforts to implement MOVE! at the facilities with high implementation effectiveness. The importance of management support has broad backing in the literature [28]. This finding is directly confirmed by a study of another widely disseminated complex program in VA aimed at reducing waiting times at primary care and specialty clinics in which a quantitative measure of management support was one of only two significant predictors of effective implementation [29]. In Klein, Conn, and Sorra's implementation model, management support has a direct effect on implementation policy and practices. The more committed managers are to the implementation, the more likely they will provide the resources and support needed e.g., training, space, communications. Our data support the existence of this relationship. Managers at the high-implementation and transition facilities helped to establish supportive practices and infrastructure by increasing program visibility, dedicating staff time to MOVE!, making it clear to service chiefs that participation of their staff in MOVE! was expected, and provided active and moral support to frontline staff.

More active management support could be encouraged by explicitly defining roles and responsibilities for different levels of management to implement a complex program like MOVE!. In the absence of management support, local champions could help fill the void to some extent [30]. We described one example of a physician champion at a facility with low implementation effectiveness who together with the above-and-beyond efforts by the MOVE! coordinator, succeeded in launching a program in a challenging context. It was striking that at three of the five facilities, little or no mention was made of their physician champions, even when asked directly. Thus, the importance of role of champion was mixed and may be compensated for by the positive presence of other constructs. It appeared that some of the physician champions were appointed into the role by facility leadership. It is important, instead, to identify champions who truly believe in the program, are open to change, are respected by their peers, have good communication and leadership skills, and willingly volunteer to take an active role by promoting the program broadly in their organization, serving as a local expert, using their influence to persuade peers to support and engage with the program - all this for the duration of time it takes to sustain skilled, enthusiastic use of the program [30,31].

Four of the five study facilities provided initial assessments and a program of group visits but none of the facilities provided consistent phone-based self-management support, which is a foundational treatment component of MOVE!. Staff at our study facilities did not have sufficient time nor did they feel confident in their ability to help Veterans over the phone. As a result of this latter finding, NCP is piloting a national call center to provide self-management support for Veterans. In addition, a home-based "TeleMOVE" program was implemented in FY2010 that allows Veterans to receive daily motivational messages through a home-monitoring device, "checking-in" with weekly weights, and requesting a call from program staff when needed. These alternative programs overcome facility barriers related to space, logistical and transportation barriers to patients attending facility-based programs, and in the case of TeleMOVE, allows clinicians to monitor a large patient panel with an automated intervention.

The influential role of some implementation policies and practices, as defined by Klein and colleagues [17], was supported by our findings. The most frequently mentioned need was dedicated time. Staff at the two facilities with low implementation effectiveness did not have explicitly dedicated time allocated to MOVE!. At the transition facility and facilities with high implementation effectiveness, winning formal approval for dedicated time was a key enabler of their success. Once dedicated time was approved, the MOVE! coordinator, especially, was able to do the legwork necessary to negotiate for space and other resources needed to implement the program. Our qualitative findings also revealed the critical role communication played in making MOVE! visible within the organization [32]. In addition to specific communications about MOVE! however, there is also support for expanding the definition to include the role communications play in coalescing teams and building a shared vision [32]. MOVE! staff at high implementation facilities described a more cohesive team of committed staff, despite the fact that each team member reported to a separate supervisor in separate units. There is support for coalescing multi-disciplinary teams [33] contributing to successful implementation [34]. These teams displayed an assertive, problem-solving approach to overcoming issues. This was in contrast to staff at the low-implementation facilities who did not meet face-to-face but rather communicated mostly through email.

Implementation climate, reflecting the importance of implementing MOVE!, was higher at facilities with a high level of implementation effectiveness. We described the challenge of implementation in a facility struggling to reduce large backlogs in primary care clinics which was a higher priority goal for the organization than implementing a new weight management program. VHA had implemented Advanced Clinic Access which is an established set of principles designed to give Veterans access to healthcare when they need it [35]. Pressure to meet performance measures related to backlogs in primary care clinics was strong. We heard how, at one of the facilities, providers were working through lunch and on weekends to get their backlog of patients down. This highly visible initiative reduced the priority placed on MOVE! implementation. Other VHA performance measures related to reducing physiologic measures among patients (e.g., blood pressure) in FY2007 also seemed to work against MOVE! implementation efforts at one facility with low implementation effectiveness. Clinical leaders did not believe lifestyle change, as embodied by MOVE!, would impact these measures and thus were not willing to allocate the resources necessary to implement the program. A performance measure was implemented to promote screening for overweight/obesity and referrals to MOVE! at the start of FY 2009 [36]. Over the course of two years (FY2009-2010), the number of patients screened and asked about their willingness to be referred to MOVE! increased from 79% to 96% system wide. This increase is likely to have increased priority for implementing MOVE! at local facilities [37]. Establishing *clinical *priority for the program is also important. Providers at the two high-implementation facilities understood and articulated the role of MOVE! in helping their patients lose weight as a strategy to reduce the incidence or severity of obesity-related chronic conditions.

The qualitative findings supported our quantitative data, both of which validated our theoretical framework by highlighting significant differences in the contextual factors between the high and low implementation facilities. Management support, implementation policies and practices (communications and sufficient time), the fit of MOVE! with values and existing programs, and implementation climate were all qualitatively different and quantitative measures of these constructs were rated more positively at the high compared to the low implementation facilities. Furthermore, scores for the transition facility fell between low- and high-implementation facilities, reflecting the initial difficulties they had in getting the program implemented tempered by their more recent success in getting a viable program off the ground. The only construct that did not appear to distinguish between low and high implementation facilities was the role of physician champions, suggesting that champions alone are insufficient for overcoming other important barriers.

Thus, the quantitative measures appear to be reasonable indicators of the strength of influence of organizational factors in getting MOVE! implemented. The obvious benefit of the quantitative data is their ease and efficiency of administration, in contrast to the data obtained from the open-ended interview questions, which are particularly time consuming to code and analyze. Nevertheless, the qualitative data provide insight into exactly how each construct served as a barrier or facilitator to implementation effectiveness, thus providing the data necessary for making recommendations for future implementation efforts.

Several study limitations merit consideration and provide context for interpreting our findings. First, this was a cross-sectional study of a small number of purposively selected facilities and thus generalizability is limited. However, our goal in this study was to more deeply understand contextual factors facilitating or hindering initial implementation of MOVE! and how those factors differ between high and low-implementation facilities. A purposive sampling design was selected to maximize variation to identify important differences and potential common patterns across a diverse sample of medical centers. Second, the investigators, coders, and analysts together, assessed implementation of MOVE! treatment components and were thus aware of the status of implementation at each facility. This knowledge has potential to bias qualitative findings. However, the diversity of the team, its confirmation by quantitative findings, and our careful analytic approach helped to minimize this potential bias. Third, it is important to note that our findings were based on data collected in the second year after MOVE! was disseminated (18-22 months later) and do not reflect MOVE! implementation today. Lessons learned from this experience, however, can benefit other large dissemination efforts. Fourth, though we used a theoretical model to guide our data collection and analyses and our findings are promising, our sample of five facilities was not sufficient to validate the model for this setting. We had insufficient data to confirm the mediating roles of individual constructs in the model. Instead, we simply examined the presence or absence of each of the constructs, rather than their relationships with each other as depicted in Figure 1. Further research is needed across multiple studies to examine these relationships.

## Conclusions

Key organizational factors at local VHA medical centers were strongly associated with MOVE! implementation. Findings point to recommendations that can help accelerate large-scale dissemination of complex lifestyle behavior change programs, including recommendations related to the role of managers, champions, sufficient resources, and communications within multi-disciplinary teams.

## Competing interests

The authors declare that they have no competing interests.

## Authors' contributions

LJD made substantial contributions to conception and design, acquisition of data, and analysis and interpretation of data and was involved in drafting the manuscript. DEG made substantial contributions to analysis and interpretation of data and was involved in drafting the manuscript. CHR made substantial contributions to analysis and interpretation of data and was involved in drafting the manuscript. CEF made substantial contributions to analysis and interpretation of data and reviewed manuscript drafts. JCL made substantial contributions to conception and design and analysis and interpretation of data and reviewed manuscript drafts. All authors read and approved the final manuscript.

## Endnotes

i Participants are Veterans who received at least one MOVE! visit during the FY. The rate of participation of Veterans was 20.72 participants per 1000 Veterans. The number of MOVE! candidate Veterans was estimated based on an average prevalence of 65% of Veterans who are candidates for MOVE! (20.72/0.65 = 28.78). Personal communication with Dr. Leila Kahwati at NCP in April 2011.

## Pre-publication history

The pre-publication history for this paper can be accessed here:

http://www.biomedcentral.com/1472-6963/11/248/prepub

## Supplementary Material

Additional file 1 **Interview Guide**. Interview guide including close-ended, quantitative questions, followed by open-ended qualitative questions. Not all questions were asked of all participants. Click here for file

## References

[B1] Flegal KM Carroll MD Ogden CL Curtin LR Prevalence and Trends in Obesity Among US Adults, 1999-2008 JAMA 2010 2009 2014 10.1001/jama.2009.2014 20071471

[B2] Nelson KM The burden of obesity among a national probability sample of veterans J Gen Intern Med 2006 21 9 915 919 10.1007/BF02743137 16918734 PMC1831589

[B3] VA Benefits & Health Care Utilization http://www1.va.gov/VETDATA/Pocket-Card/4X6_spring10_sharepoint.pdf

[B4] Adams KF Schatzkin A Harris TB Kipnis V Mouw T Ballard-Barbash R Hollenbeck A Leitzmann MF Overweight, obesity, and mortality in a large prospective cohort of persons 50 to 71 years old N Engl J Med 2006 355 763 778 10.1056/NEJMoa055643 16926275

[B5] Pardo Silva MC De Laet C Nusselder WJ Mamun AA Peeters A Adult obesity and number of years lived with and without cardiovascular disease Obesity Silver Spring, Md 2006 14 1264 1273 10.1038/oby.2006.144 16899808

[B6] Wyatt S Winters K Dubbert P Overweight and obesity: prevalence, consequences, and causes of a growing public health problem Am J Med Sci 2006 331 166 174 10.1097/00000441-200604000-00002 16617231

[B7] Sullivan PW Ghushchyan VH Ben-Joseph R The impact of obesity on diabetes, hyperlipidemia and hypertension in the United States Qual Life Res 2008 17 8 1063 1071 10.1007/s11136-008-9385-7 18777200

[B8] Chenoweth D The Economic Cost of Physical Inactivity and Excess Weight in American Adults Journal of the International Society for Physical Activity and Health 2006 3 2 10.1123/jpah.3.2.148 28834464

[B9] Tsai AG Williamson DF Glick HA Direct medical cost of overweight and obesity in the USA: a quantitative systematic review Obes Rev 2010 10.1111/j.1467-789X.2009.00708.x PMC2891924 20059703

[B10] Kinsinger LS Jones KR Kahwati L Harvey R Burdick M Zele V Yevich SJ Design and dissemination of the MOVE! Weight-Management Program for Veterans Preventing chronic disease 2009 6 3 A98 19527600 PMC2722407

[B11] National Institutes of Health Clinical Guidelines on the Identification, Evaluation, and Treatment of Overweight and Obesity in Adults: The Evidence Report 1998 NIH Publication No. 98-4083

[B12] North American Association for the Study of Obesity The practical guide: identification, evaluation, and treatment of overweight and obesity in adults 2000 Bethesda, MD: National Institutes of Health; National Heart, Lung, and Blood Institute Volume NIH Publication no. 00-4084

[B13] US Preventive Services Task Force Screening for obesity in adults: recommendations and rationale Ann Intern Med 2003 139 11 930 932 14644896 10.7326/0003-4819-139-11-200312020-00012

[B14] VA/DoD Clinical Practice Guideline for Screening and Management of Overweight and Obesity Version 1.0 http://www.oqp.med.va.gov/cpg/OBE/OBE_base.htm

[B15] Creswell JW Clark VLP Designing and Conducting Mixed Methods Research 2007 1 Thousand Oaks, CA: Sage Publications, Inc

[B16] Klein KJ Sorra JS The Challenge of Innovation Implementation The Academy of Management Review 1996 21 4 1055 1080

[B17] Klein KJ Conn AB Sorra JS Implementing computerized technology: An organizational analysis J Appl Psychol 2001 86 5 811 824 11596799 10.1037/0021-9010.86.5.811

[B18] Helfrich CD Weiner BJ McKinney MM Minasian L Determinants of implementation effectiveness: adapting a framework for complex innovations Med Care Res Rev 2007 64 3 279 303 10.1177/1077558707299887 17507459

[B19] Miles MB Huberman AM Qualitative Data Analysis: A Sourcebook 1994 2 Thousand Oaks, CA: Sage Publications

[B20] Belli RF The structure of autobiographical memory and the event history calendar: potential improvements in the quality of retrospective reports in surveys Memory 1998 6 4 383 406 10.1080/741942610 9829098

[B21] Forman J Damschroder LJ Jacoby L, Siminoff L Qualitative Content Analysis Empirical Research for Bioethics: A Primer 2008 11 Oxford, UK: Elsevier Publishing 221

[B22] Hill CE Knox S Thompson BJ Williams EN Hess SA Consensual Qualitative Research: An Update Journal of Counseling Psychology 2005 52 2

[B23] Hill CE Thompson BJ Williams EN A guide to conducting consensual qualitative research The Counseling Psychologist 1997 25 517 572 10.1177/0011000097254001

[B24] Sandelowski M Barroso J Writing the proposal for a qualitative research methodology project Qual Health Res 2003 13 6 781 820 10.1177/1049732303013006003 12891715

[B25] Hill CE Thompson BJ Williams EN A guide to conducting consensual qualitative research The Counseling Psychologist 1997 25 4 517 572 10.1177/0011000097254001

[B26] Patton MQ Qualitative research and evaluation methods 2002 3 Thousand Oaks, CA: Sage Publications

[B27] StataCorp Intercooled Stata 11 2007 College Station, Texas: StataCorp LP

[B28] Damschroder L Aron D Keith R Kirsh S Alexander J Lowery J Fostering implementation of health services research findings into practice: a consolidated framework for advancing implementation science Implement Sci 2009 4 1 50 10.1186/1748-5908-4-50 19664226 PMC2736161

[B29] VanDeusen Lukas CV Meterko MM Mohr D Seibert MN Parlier R Levesque O Petzel RA Implementation of a clinical innovation: the case of advanced clinic access in the Department of Veterans Affairs J Ambul Care Manage 2008 31 2 94 108 18360170 10.1097/01.JAC.0000314699.04301.3e

[B30] Damschroder LJ Banaszak-Holl J Kowalski CP Forman J Saint S Krein SL The role of the champion in infection prevention: results from a multisite qualitative study Quality & safety in health care 2009 18 6 434 440 10.1136/qshc.2009.034199 19955453

[B31] Kirchner JE Parker LE Bonner LM Fickel JJ Yano EM Ritchie MJ Roles of managers, frontline staff and local champions, in implementing quality improvement: stakeholders' perspectives J Eval Clin Pract 2011 10.1111/j.1365-2753.2010.01518.x 20738467

[B32] Pearce CL Ensley MD A reciprocal and longitudinal investigation of the innovation process: the central role of shared vision in product and process innovation teams (PPITs) Journal of Organizational Behavior 2004 25 2 259 278 10.1002/job.235

[B33] Iedema R Meyerkort S White L Emergent modes of work and communities of practice Health Serv Manage Res 2005 18 1 13 24 10.1258/0951484053051906 15807977

[B34] Shortell SM Marsteller JA Lin M Pearson ML Wu SY Mendel P Cretin S Rosen M The role of perceived team effectiveness in improving chronic illness care Med Care 2004 42 11 1040 1048 10.1097/00005650-200411000-00002 15586830

[B35] Schall MW Duffy T Krishnamurthy A Levesque O Mehta P Murray M Parlier R Petzel R Sanderson J Improving patient access to the Veterans Health Administration's primary care and specialty clinics Jt Comm J Qual Saf 2004 30 8 415 423 15357131 10.1016/s1549-3741(04)30047-x

[B36] 2008 highlights - MOVE! http://www.prevention.va.gov/2008_Highlights_MOVE.asp

[B37] VanDeusen Lukas CV Holmes SK Cohen AB Restuccia J Cramer IE Shwartz M Charns MP Transformational change in health care systems: An organizational model Health Care Manage Rev 2007 32 4 309 320 18075440 10.1097/01.HMR.0000296785.29718.5d

