# Supplementary material for: A systematic exploration of differences in contextual factors related to implementing the MOVE! weight management program in VA: A mixed methods study
Source: BMC Health Serv Res. 2011 Sep 30;11:248. doi: 10.1186/1472-6963-11-248 (PMC3206421; doi:10.1186/1472-6963-11-248)
Supplement: Additional file 1 — Interview Guide. Interview guide including close-ended, quantitative questions, followed by open-ended qualitative questions. Not all questions were asked of all participants. [file 1472-6963-11-248-S1.DOC]

Hello, my name is [name of interviewer]. *Introduce self and other team members in the room.*

- Introduce the project

As you know, the purpose of this Managing Overweight/Obesity for Veterans Everywhere (*MOVE!*) implementation study is to better understand barriers and facilitators for implementing *MOVE!* with the intent to improve implementation of the current program and as pre-implementation work for a future enhanced *MOVE!* implementation study. We want to take this time to talk to you about your experience, so far, with the *MOVE!* program. Your input will help us to evaluate *implementation and variation* in the *MOVE!* program within and between facilities. We want to understand the challenges and successes of adopting and implementing MOVE! so that we can maximize the likelihood that other sites will be successful with this program.

- *Explain the purpose of the interview*

Your interview will help us to better understand the challenges and successes of the *MOVE!* program at your facility. We will be interviewing multiple people at your facility to gain multiple perspectives. We are really interested in learning more about your own experience with this program.

We are going to ask you questions about your experience <*role e.g., MOVE! Site Coordinator*> for your facility.

- *Describe the audio recording and how we will assure confidentiality and answer any questions*

This interview will be audio taped so that we have an accurate record of your thoughts. Please be assured that the tapes and your transcript will be kept confidential. Leadership at your facility, any other co-workers, or NCP leadership will not have access to any of your responses nor be able to connect your responses to you personally. Our study does have Ann Arbor IRB approval. Once your interview has been transcribed, only a site identifier will be linked to the transcripts, while any information linking you to the transcript will be destroyed. The audio recording will be destroyed as soon as the transcript is verified and analyzed by research staff.

To help ensure confidentiality, it would help if you would refrain from mentioning the name of your facility wherever possible or mentioning specific names of other staff members during the interview. If, at any time, you feel that the questions are too sensitive, I would be happy to turn off the recorder during that portion of questioning. You may also skip any questions you wish during the interview.

We also wanted to remind you, that we will giving you a $10 Target gift card as a small token of our appreciation for your participation.

Do you have any questions for me? [Answer any questions]

Are you ready to begin? I’m going to start recording now.

**I. INTRODUCTION**

I’m going to begin by asking a short series of closed-ended questions. There is no right or wrong answer to any of these questions. The possible responses for each item are: 1-strongly disagree, 2-disagree, 3-neutral, 4-agree, 5-strongly agree, or unknown/not applicable. Please give the response that fits most closely with your perception.

*The following quantitative items are adapted from:Klein KJ, Conn AB, Sorra JS: Implementing computerized technology: An organizational analysis. J Appl Psychol 2001, 86(5):811-824.*

First, I would like to talk about COMMUNICATIONS to staff about MOVE! in your facility:

Please tell me how much you agree or disagree with each statement.

1. I have access to a lot of information about MOVE!.

2. Staff here are well informed about MOVE!.

3. Staff here often feel like they are being kept in the dark about MOVE!

4. Staff understand the reasons why this VAMC is implementing MOVE!.

5. I have been kept informed about information related to MOVE!.

6. Staff know the specific goals that this VAMC hopes to achieve by implementing MOVE!.

Now, I would like to talk about the general CLIMATE related to MOVE! at your facility:

Please tell me how much you agree or disagree with each statement.

1. MOVE! is a top priority at my facility.

2. MOVE! takes a back seat to other programs at my facility.

3. People put a lot of effort into making MOVE! a success here.

4. People at my facility believe that implementation of MOVE! is important.

5. An important goal at my facility is to implement MOVE! effectively.

6. People here really don't care about the success of MOVE!.

7. There is a big push for people to make the most of MOVE! at my facility.

Now, I would like to talk about MANAGEMENT SUPPORT for MOVE! at your facility:

Please tell me how much you agree or disagree with each statement.

1. Since *MOVE!* started at this hospital, managers and supervisors have actively pushed to make *MOVE!*  a success.

2. Managers and supervisors are strongly committed to the successful implementation of *MOVE!*

3. Managers and supervisors have expressed doubts about whether *MOVE!*  will really help patients at this hospital.

4. Managers and supervisors show little interest in *MOVE!*

5. Managers and supervisors stress the importance of *MOVE!*  for this hospital.

1. Managers and supervisors take an active interest in *MOVE!* -related problems and successes.

Now, I would like to talk about TIME AVAILABILITY for MOVE! –related activities:

Please tell me how much you agree or disagree with each statement.

1. People at my facility feel that they have enough time to do their work and to learn new skills associated with delivering *MOVE!* -related services.

2. Most people are so busy that they have very little time to devote to the implementation of *MOVE!*

1. Employees are encouraged to take time off from their regular work tasks to attend

*MOVE!*  meetings and training sessions.

Lastly, I would like to talk about RESOURCES that have been made available for implementing MOVE! at your facility/VISN. Please tell me how much you agree or disagree with each statement.

1. Financial constraints at my facility/VISN have made it difficult to offer as much staff education and training about the *MOVE!* program as needed.

2. Because of financial constraints at my facility/VISN, staff members involved with MOVE! have been unable to devote as much time as needed to implementation the program in for area.

3. Pressures from hospital management caused people to rush ahead with implementation of *MOVE!*  before my facility/VISN really was ready.

4. Resources have been readily available to support activities related to the implementation of *MOVE!* at my facility/VISN.

5. We have had to implement *MOVE!*  with very limited resources.

6. My facility has been unable to provide adequate resources to provide clinical support for MOVE! including, referrals, assessments, counseling, (phone or on-site visits), and educational activities needed to implement *MOVE!* effectively.

1. Adequate resources are available to support *MOVE!*  implementation efforts at my facility.

We will turn now to the main part of the interview where our questions will be open ended. We want to hear your thoughts so please do not hesitate to share whatever you believe might be related to any of the topics.

<Note: Questions *were selected from the following list based on the role of the participant, on-going findings as the interviews progressed, and other contextual factors.>*

**I would like to ask you a few questions to help me understand your role and who was involved with *MOVE!* at your site.**

**Will you please describe your role within your facility?**

*PROBES*

- What is your title and role within your organization?
- Who do you report to?
- What is your role in the *MOVE!* program?

**II. OPEN-ENDED DESCRIPTION OF IMPLEMENTATION**

**Now, I would like to hear about the process you went through to implement the *MOVE!* intervention. Later, in the interview, we will talk about particular parts in more detail. For now, though, will you please describe how *MOVE!* was implemented at your site, to the best of your recollection? If you can, please tell me about the major milestones and the month and year in which they each occurred.**

*PROBES*

- Use any background information that might be available in terms of milestone dates, etc.
- Are they offering telephone or individual follow-visits (Level 1)? MOVE! Classes (Level 2)?

**III. PRE-IMPLEMENTATION EXPERIENCES**

**I would like to hear more about how the intervention initially started at your site and how you and others viewed the *MOVE!* program before it was implemented.**

**To what degree did people involved with *MOVE!*, buy-in to the intervention before it was implemented? Why?**

**Did you see a need for this type of intervention? Why or why not?**

*PROBES*

- At that time, how did you think *MOVE!* would meet that need for clinicians? To what extent did it *actually* meet that need?
  - Improvement in work process (amount or type)?
- …how did you think *MOVE!* would meet that need for patients? To what extent did it *actually* meet that need?
  - Better patient outcomes? Which outcomes?

**What kind of services were you already offering to patients who are overweight or obese at your site?**

*PROBES*

- - To what extent were the existing services multi-disciplinary ?
- How were patients being treated?
- To what extent?
- Were there other similar initiatives?
- Do you perceive MOVE as being a superior alternative? Why or why not?
- To what degree was there “competition” for funds, time, or attention because of other initiatives that may have been taking place concurrently?

**IV. IMPLEMENTATION EXPERIENCES**

**Resource Availability**

**What type and how much IT support did you have when implementing *MOVE!*?**

**Has this sustained over time?**

*PROBES*

- Examples (EMR support, *MOVE23!* Patient Questionnaire support)
- Did you encounter technical issues? How were they resolved?
- How is workload reported? Credit?
  - - (Note, the following codes are supposed to be used to report MOVE! workload: 372 Individual Visit; 373 Group Visit; 324+372 or 147+372 telephone. Must be set up as a “count” clinic (not noncount) and must all be “checked-out”

**Will you please describe the physical space configuration used for *MOVE!*?**

*PROBES*

- What kind of space was used for patient interviews and visits? Group Visits?
- Where were you located (relative to others working on *MOVE!*)?

**VI. OPERATIONAL EXPERIENCES**

**Resource and Time Availability**

**Once *MOVE!* was fully implemented and you started referring patients, how much time and effort was/is required to provide MOVE! on a day-to-day basis?**

*PROBES*

- Was additional or unforeseen time or effort incurred in getting everything implemented? E.g., IT support, trouble-shooting equipment, etc.
- What resources would be needed to keep *MOVE!* going on a long-term basis?

***EXTERNAL CONTEXT***

**Communications**

**What disciplines or services are involved with MOVE! at your facility?**

*PROBES*

- Dietitians only? Physicians (which specialties)? Other?
- Describe their involvement.

**Resource Availability**

**Will you please describe the process you used to schedule patients for *MOVE!* visits?**

*PROBE*

- What kind of support did you have from IT?
- What kind of support did you have from others? for example, supervisors and support for allocating appointment slots to *MOVE!* patients

**Management Support**

**What kind and level of involvement do leaders at your facility have with the *MOVE!* program?**

*PROBES*

- What kind of support did they give you? Specific example.
- Did they know about the *MOVE!* study?

**Resource Availability**

**What kind of funding support did you receive for participating in *MOVE!*?**

*PROBES*

- FTE support?
- Money?
- Relief from other duties?
- Were there resources or support that would have helped you implement *MOVE!*, that you didn’t receive?

**Did you have any funding constraints while getting *MOVE!* implemented?**

*PROBES*

- Inadequate staffing?
- Inadequate time?
- Competing priorities?

**VIII. OVERALL EXPERIENCE**

**I’d like to switch gears a bit and ask some more general questions.**

**Management Support**

**If facility-level: What kind of external support did you receive from your VISN?**

**If VISN-level: What kind of external support did you receive from the National Center for Health Promotion and Disease Prevention Program office, the VA Central Office responsible for MOVE!?**

**During Implementation? Since then?**

*PROBES*

- Talk about support in terms of mentoring, problem solving, training
- Ask for specific examples.
- Did you have adequate communications with study staff? Externally? Internally?
- Did you feel adequately supported by study staff? Externally? Internally?
- If VISN-level: Are the bi-monthly meetings with NCP helpful? Why or why not?
- *What about MOVE! Program materials distributed via Facility Toolkits and available on the MOVE! Intranet Website:* Examples, the *MOVE!* Clinical Reference Manual, the *MOVE!* Quick Start Manual, the *MOVE!* Pocket guides, *MOVE!* Reference Tools, the Ten Minute *MOVE!* Staff Orientation Video.
- How helpful was this information?

**Communications**

**Generally speaking, what do clinicians at your facility think of the *MOVE!* program?**

***Time Availability***

***Clinicians:* Even without *MOVE!*, do you feel like you are able to get everything done you would like to in a typical day? Why or why not? Is this true for the other clinicians in your unit? Why or why not?**

*PROBE*

- Do you have other competing priorities?
- What is the nature of those conflicts?
- Short term? Long term?

***Communications***

***Clinicians only:* How would you describe working relationships between units/services at your facility?**

*PROBES*

- How empowered do clinicians feel in doing their work? Interacting with *MOVE!*?
- Do clinicians have close, high-performing relationships?
- How were the relationships between clinicians and IT or other hospital administrative staff or support staff (ie. ADPACs, DSS people, MAS people, clerks, facilities managers (scheduling group rooms, etc.)?
- How do these relationships affect the ability to refer patients, schedule appointments, conduct the interviews, setting things up, etc

**Some people rely more on formal communication (e.g., email communications through the chain-of-command) while others use informal communication (e.g., a hallway conversation with a co-worker) to accomplish things. Which type of communication tends to be most helpful for you to get things done?**

*PROBE*

- Focus on informal communications that include unscheduled discussions between staff outside of formal meetings.
- Were the informal communications helpful?
  - If there was not much informal communication, why not? Was the lack of informal communication a detriment?
- Ask for examples related to *MOVE!*.

**IX. LEVEL OF ENDORSEMENT AND RECOMMENDATIONS**

**On a scale of 0-10, how successful do you think *MOVE!* is at your site? Why?**

Note: We are interested in the “perception” of success; the interviewee can define success in any way.

**If you had the option, would you recommend continuing *MOVE!* at your site? Why or not?**

*PROBES*

- What would it take to keep *MOVE!* going at your site?
- What type of justification would you need to show in order to keep this intervention going over the long-term?

**Would you recommend *MOVE!* to other sites? Why or why not?**

**Do you have any specific suggestions for other sites who have not yet started implementing *MOVE!*?**
